# Cardiac involvement in cancer patients under chemotherapy and diagnosed with COVID-19: case report and literature review

**DOI:** 10.11604/pamj.2022.41.45.23951

**Published:** 2022-01-18

**Authors:** Hanane Mechal, Rime Benmalek, Hamza Choukrallah, Anas Maaroufi, Rachida Habbal, El Ghali Mohamed Benouna, Anass Mounir, Khalid Allali, Rachid Cherkab, Lhoucine Barrou, Chafik El Kettani

**Affiliations:** 1 COVID-19 Dedicated Cardiology Team, University Hospital Center of Casablanca, Morocco,; 2 COVID-19 Dedicated ICU Team, University Hospital Center of Casablanca, Morocco

**Keywords:** COVID-19, cardiac injury, chemotherapy, anthracyclines, case report

## Abstract

Many cases of severe cardiac complications due to Coronavirus disease 2019 (COVID-19) were reported. Cancer and chemotherapy appear to be risk and prognostic factors for COVID-19. A 49-year-old Female, with a history of breast cancer treated by tumorectomy and anthracycline-based chemotherapy was admitted with acute respiratory distress syndrome (ARDS) confirmed as COVID-19. She also had elevated troponin I level (up to 43 g/L), and diffuse myocardial hypokinesia along with severe left ventricle dysfunction on echocardiography. Initial treatment included hydroxychloroquine, azithromycin, corticosteroids and mechanical ventilation. The evolution was marked by QT interval prolongation (QTc=523 ms) and occurrence of cardiogenic shock. The patient died of hemodynamic instability reluctant to resuscitation measures at the 2^nd^day of hospitalization. COVID-19 patients may develop severe cardiac complications such as myocarditis and heart failure. Receiving chemotherapy especially anthracyclines may be a precipitating and prognostic factor of cardiac manifestations in COVID-19 cancer patients.

## Introduction

The coronavirus disease 2019 (COVID-19) due to the Severe acute respiratory syndrome coronavirus 2 (SARS-CoV-2), has rapidly grown into a pandemic. Its manifestations are mainly respiratory and can result in severe forms responsible for an Acute Respiratory Distress Syndrome (ARDS) requiring admission to an Intensive Care Unit (ICU). On the other hand, many cases of cardiac complications including acute myocarditis, acute coronary syndrome, arrhythmias, venous thromboembolism and heart failure, were reported [1]. COVID-19 may either induce new cardiac pathologies and/or exacerbate underlying cardiovascular diseases. Vulnerable patients include old age, medical comorbidities, immunodeficiency, etc. These characteristics are also common in cancer patients. The severity of cardiovascular effects of COVID-19, along with the side effects of specific treatments is not known yet in the population of cancerous patients, and is subject to close investigation. Several reports are beginning to emerge. The aim of our report was to describe cardiac complications in cancer patients receiving anthracycline-based chemotherapy and diagnosed with COVID-19.

## Patient and observation

We report the case of a 49-year-old female, without any cardiovascular risk factors or any cardiovascular disease history. She had a history of breast cancer treated by tumorectomy and anthracycline-based chemotherapy, receiving a total of four Doxorubicin sessions with a cumulative dose of 240mg/m^2^, the last cure was received 10 days before her hospitalization.

**Figure 1:**
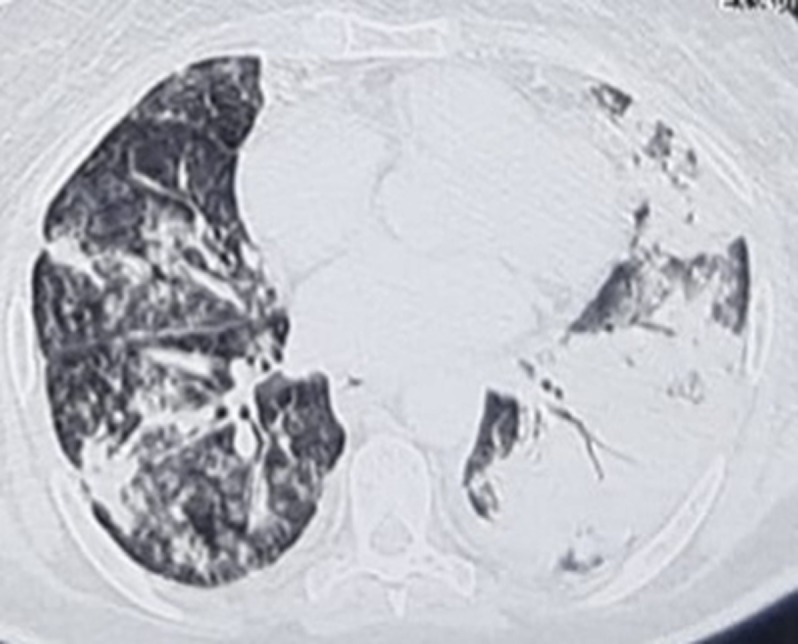
thoracic CT scan showing bilateral extended alveolar interstitial infiltrates exceeding 75% in the left side in favor of COVID-19

**Figure 2:**
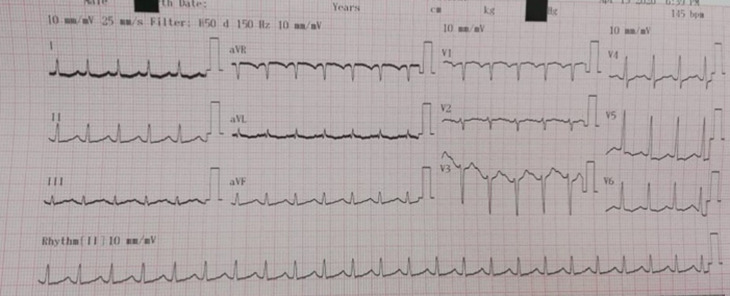
electrocardiogram at admission showing sinus tachycardia with narrow QRS-complex and a normal corrected QT interval

**Figure 3:**
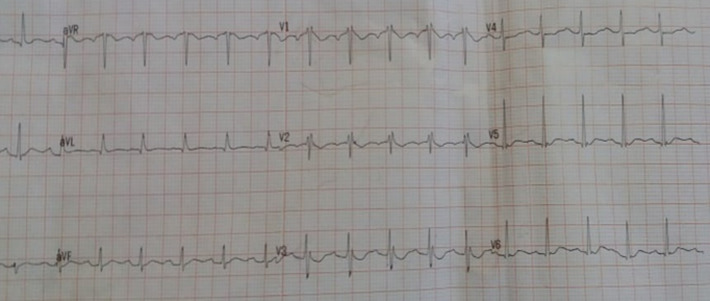
electrocardiogram at 2^nd^ day of hospitalization showing QTc interval prolongation (up to 523 ms) and QRS widening (right bundle branch block)

The patient was admitted for ARDS in the ICU and was confirmed as having COVID-19 according to findings of diffuse ground glass opacities in thoracic Computed Tomography (CT) scan evoking a viral pneumonia (Figure 1) and positive testing of nasopharyngeal samples. Initial clinical examination in the ICU revealed a patient in respiratory distress, with an oxygen saturation of 85% while breathing ambient air, a systolic blood pressure of 110 mmHg, a diastolic blood pressure of 70 mmHg and a heart rate of 150 bpm. Electrocardiogram at admission showed a sinus tachycardia with narrow QRS-complex and a normal corrected QT (QTc) interval (Figure 2). At admission, she was classified at Low risk of QTc prolongation accordingly to the Tisdale score (score 6/21: Female gender, serum kaliemia ≤ 3.5 mEq/L and 2 ≥ QTc interval prolonging medications). Initial treatment included *hydroxychloroquine*, azithromycin, vitaminotherapy, corticosteroids, preventive anticoagulation and mechanical intubation. On the 2^nd^day of hospitalization, Respiratory and hemodynamic functions deteriorated with the occurrence of acute pulmonary edema, troponine I level increased (up to 43 g/L), Electrocardiogram evolution was marked by QTc interval prolongation (up to 523 ms) and QRS widening (right bundle branch block) (Figure 3) and Echocardiography showed diffuse myocardial hypokinesia along with a severe decreased Left Ventricular Ejection Fraction (LVEF) estimated at 25%, an acute severe mitral regurgitation and a mild pericardial effusion.

Treatment changes included introduction of vasoactive drugs (noradrenaline and dobutamine) and intravenous Furosemide as well as stopping hydroxychloroquine and azithromycin because of the QTc interval prolongation. Given the refractory cardiogenic shock, the patient was candidate to extracorporeal membrane oxygenation (ECMO) but the hemodynamic state has quickly evolved during the same day towards cardiorespiratory arrest reluctant to resuscitation measures.

## Discussion

Cancer patients seem to have a significantly higher vulnerability to COVID-19, imputable to the cancer itself and the immunodeficiency it generates, but also to cancer treatment. Recent studies [2,3] evaluated the clinical records of cancer patients diagnosed with COVID-19 in China. In comparison to the general population, cancer patients had a higher incidence of severe events, with a mortality rate that is more than ten times higher than the one reported in all COVID-19 patients in China.

Most deaths in COVID-19 were caused by ARDS; other causes of death included cardiac complications (pulmonary embolism, infection-related myocarditis and acute myocardial infarction). Myocardial injury is one of the important pathogenic features and an important prognostic factor in COVID-19. Cardiac biomarkers suggest a high prevalence of cardiac injury in hospitalized patients, multiple studies have shown increased cardiac biomarkers mainly cardiac troponins I and T in infected patients, especially those with severe disease. Myocarditis is described as another cause of morbidity among COVID-19 patients [4]. Myocardial injury in COVID-19 was explained by several mechanisms including cardiomyocytes direct damage, systemic inflammation, interferon mediated immune response, exaggerated cytokine response, myocardial interstitial fibrosis, hypoxia as well as coronary plaque destabilization [4]. However mechanisms of cardiac involvement in cancer patients receiving chemotherapy and diagnosed with COVID-19 are more complex since both anticancer treatment, COVID-19 and its specific treatments may cause cardiovascular manifestations and affect heart function.

Doxorubicin is one of the most effective anthracycline-based chemotherapy used in breast cancer, but can be complicated by both late and acute cardiotoxicity. Subsequent studies measured changes in left ventricular ejection fraction (LVEF) with anthracycline chemotherapy and confirmed a cumulative dose-dependent decrease in Left Ventricular Function (LVEF), leading to congestive heart failure. Cardiotoxicity can occur at cumulative doses of doxorubicin >350 mg/m^2^ with a sharp increase in the prevalence of heart failure at a cumulative dose of 550 mg/m^2^. Acute anthracycline cardiotoxicity is a rare and a potentially lethal complication, occurring in the form of pericarditis, arrhythmias but mostly left ventricular dysfunction. Reports suggest that the mechanism responsible for the acute cardiotoxicity may involve an inflammatory response or a stress-induced (takotsubo) cardiomyopathy [5]. Furthermore, Studies have shown that recent use of anticancer therapies (including chemotherapy, immunotherapy and radiation) in cancer patients within 14 days of COVID-19 infection is an independent predictor of death or other severe events (HR>4) [3]. Breast cancer therapies - including chemotherapy, immunotherapy, targeted therapies and radiation) - can weaken the immune system and damage cardiac and respiratory functions, increasing even more the risk of complications if they become infected by SARS-CoV-2. The immune system recovers in the majority of cases within a couple of months after completing these treatments, so those in remission and those who received breast cancer therapies in the past don't automatically have a higher risk of severe COVID-19 disease. Those findings can justify avoiding antitumor treatments causing immunosuppression or dosages decrease in case of COVID-19 infection in cancer patients [3]. The use of hydroxychloroquine alone or in combination with azithromycin for the treatment of COVID 19 was associated with QTc interval prolongation [6]. While Anthracycline chemotherapy can alone be responsible for a prolongation of QTc interval [7], its association to hydroxychloroquine and azithromycin can increase the risk of QTc prolongation even more. In fact, many reports question the safety of using hydroxychloroquine ± azithromycin as a treatment of COVID-19 in general population [6], administration of hydroxychloroquine in cancer patients receiving anthracycline-based chemotherapy can be even more questionable.

Cancer patients show deteriorating conditions and poor outcomes from the COVID-19 infection. Given those findings the management of cancer patients might be affected by the COVID-19 pandemic. Learned societies recommend procedures that ensure a balance between preventing or treating COVID-19 in cancer patients and ensuring adequate oncological care [8]. Cancer patients, especially those still receiving anticancer treatments must practice social distancing or isolation and should have vigorous screening for COVID-19 infection including testing for virus and chest radiography [9]. And because the risk of COVID-19 infection in cancer patients is higher in healthcare facilities [3], many hospitals and health professionals recommend delaying elective surgeries, screenings, and other procedures that are not considered necessary or urgent. In the same way, tele-consultations or telephone consultations are being favored when it is not essential to carry out a clinical or a follow up examination in the immediate term [8]. These tough decisions and drastic measures are being made in order to protect cancer patients from unnecessary exposure to SARS-CoV-2 and to make sure healthcare providers have the adequate resources to treat severe patients with COVID-19 [10].

## Conclusion

As the disease spreads, there is an urge to develop a risk model for cardiac complications in COVID-19 patients in order to identify and/or predict response to various treatment modalities. Cancer patients seem to have high vulnerability to COVID-19. In these patients, determining the implication of anticancer therapy or COVID-19 in causing cardiac manifestations, can be difficult and our report outlines this challenge. The mechanism of cardiac injury caused by COVID-19 needs further study, but we can guess that chemotherapy especially anthracyclines may constitute a precipitating and prognostic factor of cardiac injury in COVID-19.
